# Low-Circulating Homoarginine is Associated with Dilatation and Decreased Function of the Left Ventricle in the General Population

**DOI:** 10.3390/biom8030063

**Published:** 2018-07-30

**Authors:** Martin Bahls, Dorothee Atzler, Marcello R.P. Markus, Nele Friedrich, Rainer H. Böger, Henry Völzke, Stephan B. Felix, Edzard Schwedhelm, Marcus Dörr

**Affiliations:** 1 Department of Internal Medicine B, University Medicine Greifswald, 17475 Greifswald, Germany; marcello.markus@uni-greifswald.de (M.R.P.M.); felix@uni-greifswald.de (S.B.F.); marcus.doerr@uni-greifswald.de (M.D.); 2 German Centre for Cardiovascular Research (DZHK), Partner Site Greifswald, 17475 Greifswald, Germany; nele.friedrich@uni-greifswald.de (N.F.); voelzke@uni-greifswald.de (H.V.); 3 Institute for Cardiovascular Prevention, LMU Munich, German Centre for Cardiovascular Research (DZHK), Partner Site Munich Heart Alliance, 80336 Munich, Germany; dorothee.atzler@med.uni-muenchen.de; 4 Walther-Straub-Institute of Pharmacology and Toxicology, LMU Munich, 80336 Munich, Germany; 5 German Center for Diabetes Research (DZD), 17489 Greifswald, Germany; 6 Institute of Clinical Chemistry and Laboratory Medicine, University Medicine Greifswald, 17475 Greifswald, Germany; 7 Institute of Clinical Pharmacology, University Medical Center Hamburg-Eppendorf, 20246 Hamburg, Germany; boeger@uke.de (R.H.B.); edzard.schwedhelm@uke.de (E.S.); 8 German Centre for Cardiovascular Research (DZHK), Partner Site Hamburg/Kiel/Lübeck, 20246 Hamburg, Germany; 9 Department of Study of Health in Pomerania/Clinical-Epidemiological Research, Institute for Community Medicine, University Medicine Greifswald, 17489 Greifswald, Germany

**Keywords:** homoarginine, echocardiography, population-based

## Abstract

Low homoarginine is an independent marker of mortality in heart failure patients and incident cardiovascular events. Whether homoarginine is related with healthier cardiac structure and function is currently unclear. We used data of the population-based “Study of Health in Pomerania” (SHIP-Trend) to assess this relation. Homoarginine was measured in serum using liquid chromatography-tandem mass spectrometry. Linear regression models assessed the relation between homoarginine and several structural as well as functional parameters and N-terminal pro B-type natriuretic peptide (NTproBNP). All models were adjusted for age, sex, body mass index, and renal function. A total of 3113 subjects (median age 48 (25th percentile 37 to 75th percentile 60) years, 46% male) were included. A standard deviation decrease in homoarginine was associated with a larger left ventricular diastolic diameter (0.3; 95%-confidence interval (CI): 0.2 to 0.5 mm; *p* < 0.001), left ventricular systolic diameter (0.38; 95%-CI: −0.22 to 0.54 mm; *p* < 0.001) as well as a less relative wall thickness (–0.003 95%-CI: −0.006 to −0.0008; *p* = 0.01), left ventricular ejection fraction (–0.47; 95%-CI: –0.79 to −0.15%; *p* < 0.01) and fractional shortening (−0.35; 95%-CI: −0.62 to 0.07%; *p* = 0.01). Low homoarginine was also related to higher NTproBNP (−0.02 95%-CI: −0.034 to −0.009 log pg/mL; *p* < 0.01). Lower serum homoarginine is associated with dilatation of the heart and decreased function. Prospective clinical studies should assess if homoarginine supplementation improves cardiac health in subjects with low serum concentrations.

## 1. Introduction

l-arginine (Arg) is a semi-essential amino acid with a vital role in living organisms, being part of several metabolic pathways [1]. For example, Arg is a substrate for the synthesis of nitric oxide (NO) by NO synthase (NOS). In addition, arginase catalyzes the conversion of Arg to ornithine, keeping the urea cycle in motion. Further, arginine:glycine amidinotransferase (AGAT) catalyzes the formation of l-homoarginine (hArg) from Arg [2]. Even though hArg may serve as an alternative substrate for NO and inhibits arginase in a similar extend compared to lysine, its low physiological concentration and affinity to enzymes make hArg unlikely to play a significant role in the physiology of the NO pathway [3,4].

Nevertheless, recent experimental and clinical data indicate a potential role of hArg in cardiovascular disease (CVD), since hArg deficiency or impairment is associated with adverse outcomes [5,6,7,8]. Specifically, low hArg was related with myocardial dysfunction and a higher risk of CVD mortality in patients referred to coronary angiography [9], patients with ischemic stroke [8], elderly subjects from the general population [10], as well as in a large German cohort with dialysis patients [5,11]. Low serum hArg concentrations were also related to an increased risk for all-cause and CVD mortality. In patients with chronic heart failure circulating hArg was associated with biochemical (N-terminal pro B-type natriuretic peptide [NTproBNP]) as well as clinical characteristics (NYHA classification) of heart failure. Furthermore, low hArg was an independent marker of all-cause mortality in this cohort [12].

Very few clinical studies have explored the relation between circulating hArg and left ventricular cardiac remodeling. Results from the “Diagnostic Trial on Prevalence and Clinical Course of Diastolic Dysfunction and Diastolic Heart Failure” (DIAST-CHF) study showed that low hArg was related with higher NTproBNP and presence of diastolic dysfunction [6]. We aimed to extend the current knowledge by assessing the relation between left and right ventricular structural and functional parameters with hArg in a general population-based cohort from northeast Germany.

## 2. Materials and Methods

### 2.1. Study Population

The presented data were derived from the population-based cohort study “Study of Health in Pomerania (SHIP) in Germany” [13]. In brief, from the total population of West Pomerania, a rural area in the northeastern part of Germany, a two-stage stratified cluster sample of 8016 adults between the ages of 20–79 years was drawn (SHIP-TREND). In total, 4420 individuals participated in the study (response of 50.1%). Data used in this analysis are based on data collected during the baseline examination of SHIP-TREND, which took place between 2008 and 2011. The study was approved by the ethics committee of the University of Greifswald, complies with the Declaration of Helsinki, and all study participants gave written informed consent. SHIP data are publically available for scientific and quality control purposes. Data usage can be applied for via www.community-medicine.de.

For the present analysis, individuals with severely impaired renal function (estimated glomerular filtration rate (eGFR) < 30 mL/min/1.73 m^2^), previous myocardial infarction, left ventricular ejection fraction < 40%, atrial fibrillation, extreme values for hArg (<0.995 percentile), and missing data were excluded. The total sample size was 3113 subjects.

### 2.2. Interview, Medical, and Laboratory Examination

Trained and certified staff used standardized computer-assisted interviews to assess sociodemographic and medical characteristics. Smoking was classified as current smoker, nonsmoker, or former smoker. Previous myocardial infarction was defined as self-reported physician’s diagnosis. Diabetic patients were identified based on the self-reported use of antidiabetic medication (anatomic, therapeutic, and chemical (ATC) code: A10) in the last 7 days or a glycosylated hemoglobin level >6.5%. All participants underwent an extensive standardized medical examination. Anthropometric measurements included height and weight based on recommendations of the World Health Organization (WHO) [14]. Body mass index (BMI) was calculated by dividing weight (kg) by height (cm) to the square. Blood pressure (BP) was assessed after a 5 min resting period in sitting position. Systolic and diastolic BP were measured three times, with three minutes rest in-between, on the right arm, using a digital blood pressure monitor (HEM-705CP, Omron Corporation, Tokyo, Japan). The average of the second and third reading was used. Hypertensive patients were identified by either self-reported antihypertensive medication (ATC: C02) or a systolic BP above 140 mmHg and/or a diastolic value of more than 90 mmHg.

Fasting venous blood samples were drawn from all subjects in supine position (between 7 am and 4 pm). The eGFR was calculated according to Levey et al. (eGFR = 186 × (plasma creatinine concentration × 0.0113118)^−1.154^ × age^−0.203^) multiplied by 0.742 for female subjects and expressed as mL/min/1.73 m^2^ [15]. Serum hArg was quantified by liquid chromatography-tandem mass spectroscopy (LC-MS/MS) analysis as previously reported [16]. Briefly, 25 μL of serum aliquots was diluted in methanol with stable isotope-labeled hArg, which was used as internal standard. Proteins were converted into their butyl ester derivatives, and subsequently subjected to mass spectrometric analysis.

### 2.3. Ultrasound Measurements

Two-dimensional, M-mode and Doppler echocardiography were performed using the Vingmed CFM 800A system (GE Medical Systems, Waukesha, WI, USA) as described in detail elsewhere [17]. Measurements of LV end-diastolic and end-systolic diameter (LVD, LVS) and septal and posterior wall thickness (SWT, PWT) were performed according to the guidelines of the American Society of Echocardiography [18]. LV mass (LVM) was calculated according to the formula: LVM (g) = 0.8 × (1.04 × ((LVDD + SWT + PWT)^3^ − LVDD^3^)) + 0.6 g as described by Devereux and Reichek [19,20]. LVM was indexed (LVMI) for body surface area (BSA) according to Duboi (BSA = 0.20247 × height (m)^0.725^ × weight (kg)^0.425^) [21], which linearizes the relations between LVM and height and identifies the impact of obesity. LV wall thickness (WT), relative wall thickness (RWT), and LV ejection fraction (EF) were calculated following the formulas below according to the guidelines of the American Society of Echocardiography [22]:

WT (cm) = (SWT + PWT)/2.

RWT = WT/LVDD.

EF (%) = (LVDV − LVSV)/LVDV.

Transmitral and transcuspid pulsed-wave Doppler were used to record early (E) and late (A) wave ventricular filling velocities. Tricuspid annular plane systolic excursion (TAPSE) is a parameter of right ventricular function. TAPSE was assessed using the four-chamber view in M-mode. Movement of the lateral tricuspid valve was measured from end-diastole to end-systole. Certification examinations for interobserver variations revealed an agreement of >90% [17].

### 2.4. Statistics

Continuous data are expressed as median and 25th/75th percentile. Nominal data are expressed as percentages. Differences between groups were calculated using Kruskal–Wallis (continuous variables) and χ^2^ test (nominal variables), respectively. First, a linear regression adjusted for sex, age, BMI, and eGFR was fitted to assess the relation between hArg and echocardiographic parameters, as well as NTproBNP continuously. Furthermore, restricted cubic splines [23] were used to detect possible nonlinear dependencies of hArg on the investigated echocardiographic parameters. Three knots were prespecified, located at the 5th, 50th, and 95th percentiles [23], resulting in one component of the spline function. A *p* < 0.05 was considered statistically significant. All statistical analyses were performed in SAS 9.4 (SAS Institute Inc., Cary, NC, USA). All continuous descriptive characteristics are presented as median and first as well as third quartile unless otherwise indicated.

## 3. Results

### 3.1. Population Description

The population characteristics are provided in Table 1. Briefly, the median age was 48 (37 to 60) years and 46% were male. Study participants had a median BMI of 26.7 (23.9 to 30.0) kg/m^2^ and an eGFR of 89 (77 to 102) mL/min/1.73 m^2^. Diabetes type 2, hypertension, and metabolic syndrome were present in 7%, 40%, and 23% of the study population, respectively. The echocardiographic parameters are grouped according to left and right ventricle, as well as structural and functional parameters.

### 3.2. Association of hArg with Left Ventricular Structural Parameters

A one standard deviation (SD) decrease in serum hArg was associated with larger LVD (0.3 95%-confidence interval [CI]: 0.2 to 0.5 mm; *p* < 0.001), LVS (0.38; 95%-CI: −0.22 to 0.54 mm; *p* < 0.001) and lower RWT (−0.003 95%-CI: −0.0008 to −0.006; *p* = 0.01). No relations were found for hArg and LVM, LVMI, PWD, left atrial, and aortic diameter (Table 2).

### 3.3. Association of hArg with Left Ventricular Functional Parameters

A one SD decrease in hArg was associated with less LVEF (−0.47 95%-CI: −0.79% to −0.15%; *p* < 0.01) and FS (−0.35 95%-CI: −0.62% to −0.07%; *p* = 0.01). No relations were found for hArg and LV diastolic functional parameters (i.e., E-wave, A-wave, A-wave duration, mitral valve deceleration time, and E/e’ ratio; Table 2).

### 3.4. Assocation of hArg with Right Ventricular Parameters

We found no association between right ventricular structural (RV and RVOT), systolic functional (TAPSE, PV acc. time, and PV acc. slope), and diastolic functional (TV S-wave, TV E-wave and TV A-wave) parameters (Table 2).

### 3.5. Association of hARG with Biochemical Parameters (NTproBNP)

**Figure 1:**
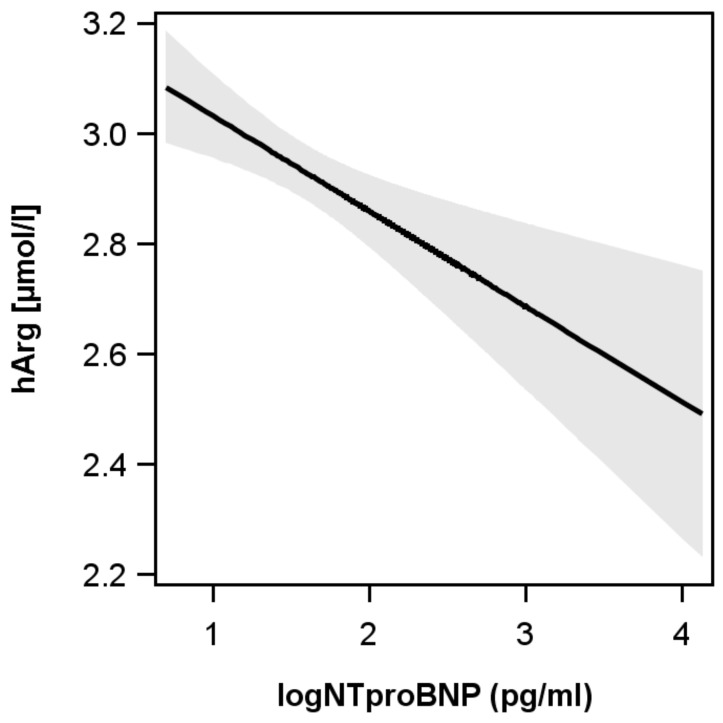
Relation between l-homoarginine (hArg) and N-terminal pro B-type natriuretic peptide (NTproBNP). Low hArg was also related to higher NTproBNP (−0.02 95%-CI: −0.034 to −0.009 log pg/mL; *p* < 0.01).

Low hArg was associated with higher NTproBNP −0.02 95%-CI: −0.034 to −0.009 log pg/mL; *p* < 0.01) (Figure 1).

## 4. Discussion

This study assessed the relation between circulating hArg and echocardiographic parameters as well as biochemical parameters of cardiac function in a large general population-based cohort. Our findings support the notion for a positive association of hArg with cardiac health. Specifically, we show that high hArg is related to lower left ventricular thickening, higher ejection fraction, and lower NTproBNP. To our knowledge, this is the first report assessing the relation between circulating hArg and a variety of echocardiographic parameters in more than 3000 subjects with preserved or midrange ejection fraction.

Our results are in agreement with previous studies with regards to the inverse association of hArg and NTproBNP. Specifically, in 3305 Caucasian patients who were referred for coronary angiography examination at a tertiary care center in Germany and recruited to the Ludwigshafen Risk and Cardiovascular Health (LURIC) study, hARG and NTproBNP were inversely associated [9]. Further support comes from the DIAST-CHF trial, in which patients with low hArg also had higher NTproBNP values [6]. However, both of these studies assessed the relation between either hArg quartiles or severity of diastolic dysfunction, respectively. Nonetheless, a study in 1255 diabetic hemodialysis patients also showed high NTproBNP concentrations in the lowest hArg quintile [11]. We extend their results by providing evidence that, even in the general population with NTproBNP values mostly below clinical cut-offs, a strong inverse association between hArg and NTproBNP is present.

With regard to left ventricular function, our results show a positive association between hArg and LVEF as well as FS. This finding is also supported by the LURIC but not the DIAST-CHF results. Specifically, LURIC patients in the lowest hArg quartile had a LVEF of 54% compared to 64% in the highest quartile [9]. Interestingly, DIAST-CHF LVEF medians were always between 61% and 62%, independent of hArg quartile [6]. The reason for this discrepancy may be related to the exclusion criteria of DIAST-CHF trial, which excluded subjects with an LVEF < 50%. In our analysis, we included midrange EF subjects and thus provided a large range of values for the regression analysis. A second different finding between our results and the DIAST-CHF analysis is related to the lack of a relation between hArg and diastolic function. However, since DIAST-CHF focused on subjects with diastolic heart failure, our subject population may not have had a sufficient number of cases to detect a relation between hArg and diastolic dysfunction.

Even though previous studies reported a relationship of hArg with LV systolic and diastolic function, there is a knowledge gap regarding subclinical LV structural remodeling. However, recent evidence from experimental mouse studies provides a first indication that low hArg may contribute to impaired *in vivo* cardiac function, suggesting a potential role in the pathophysiology of heart disease [7]. In our population-based cohort, low hArg was associated with more LV wall thickness and lower LVEF. This finding may be interpreted as low levels of hArg being related with dilatation of the heart and decreased function. Although the physiological function of hArg in the heart still remains elusive, it may be related to the well-known cardioprotective effects of NO [24]. Specifically, hArg increases NO bioavailability through at least two mechanisms. First, hArg is an alternative substrate for NOS and inhibits arginases thereby increasing l-arginine availability [3,4]. However, due to the low circulating concentrations of hArg, the physiological significance as an endogenous metabolite of the NO pathway is currently unclear [24]. Secondly, hArg may be related to cellular energy metabolism. Specifically, by its interference with AGAT, which is the first and rate-limiting enzyme of creatine synthesis. After phosphorylation, creatine acts as a spatial and temporal cellular energy buffer [25]. To date, AGAT is also considered the synthesizing enzyme of hArg in both humans and mice [2,8] and hence linking hArg to energy metabolism [7]. Interestingly, AGAT and the creatine/phosphocreatine system are downregulated in the failing heart [25,26]. A recent study assessing the AGAT knockout mice demonstrated that not low creatine, but rather low hArg was the driving force for cardiac dysfunction [7]. In our analysis, we specifically excluded subjects with heart failure and reduced ejection fraction, yet our results confirm a potentially adverse cardiac remodeling in subjects with low hArg.

Even though SHIP comprises a large and methodologically rigorous regionally representative survey of northern Germany, the findings of our analyses need to be interpreted in the context of several limitations. First, our analysis consisted of middle-aged Europeans living in rural areas. Therefore, we do not know whether our findings are also applicable to other ethnicities or age groups. Second, we used cross-sectional data from one time point. Hence, we do not know whether increases in hArg are also associated with improved left ventricular structure and function. Third, although we used a directed acyclic graph to identify metabolic and cardiovascular confounders for our multivariable models, we cannot exclude the possibility of further residual confounding. Fourth, the clinical interpretation of our results is difficult, as only ten subjects had heart failure with preserved ejection fraction. A recent Mendelian randomization analysis concluded that even though low hArg was associated with an increased risk for future hyperglycemias and abdominal obesity, there was no evidence for any causal relation [27]. Since our study was a cross-sectional association analysis, we cannot draw any causal conclusions based on our results. Hence, more experimental studies are necessary to improve our understanding of hArg biology. Irrespective of these limitations, strengths of our study are the population-based setting, the large number of individuals, the use of standardized data collection methods, the capacity to perform adjustment for a variety of clinical risk factors, and the availability of a standardized echocardiographic examination from a large number of participants.

## 5. Conclusions

Our cross-sectional analyses showed that high levels of circulating hArg in a population-based setting are associated with preferable left ventricular structure and function. We add new information with regards to LV structural remodeling. Of note, we have previously reported serum reference intervals for hArg, allowing the identification of individuals that might benefit from supplementation with hArg to normalize serum concentrations and, in addition, may support cardiac health [16]. Completed and ongoing supplementation studies in mice and humans might improve our knowledge of hArg metabolism and its mode of action [28,29].

## Figures and Tables

**Table 1:** Study population.

Parameter	Median (25th and 75th Percentile) or %
Age (years)	48 (37, 60)
Sex (% male)	45.5
Systolic blood pressure (mmHg)	125 (113, 137)
Body mass index (BMI) (kg/m^2^)	26.7 (23.9, 30.1)
Height (cm)	170 (163, 177)
Weight (kg)	77.9 (67.6, 89.2)
Smoking (%)	
Nonsmoker	37.4
Exsmoker	34.7
Smoker	27.9
Diabetes mellitus type 2 (%)	7.4
Hypertension (%)	40.5
Metabolic syndrome (%)	23.4
Estimated glomerular filtration rate (eGFR) (mL/min/1.73 m^2^)	88.5 (76.5, 102.4)
Left ventricle (LV) structural echocardiographic parameters	
Left ventricular mass (LVM) (g)	172.4 (138.4, 211.7)
LVM index (LVMI) (g/m^2^)	90.5 (76.9, 106.5)
LV diameter during diastole (LVD) (cm)	4.9 (4.5, 5.2)
LV diameter during systole (LVS) (cm)	2.9 (2.6, 3.2)
Posterior wall diameter (PWD) (cm)	1.0 (0.9, 1.1)
Relative wall thickness (RWT)	0.4 (0.3, 0.4)
Left atrium (cm)	3.8 (3.5, 4.2)
Aorta (cm)	2.8 (2.5, 3.1)
LV systolic functional echocardiographic parameters	
LV ejection fraction (LVEF) (%)	72 (66, 78)
Fractional shortening (%)	41 (36, 46)
LV diastolic functional echocardiographic parameters	
Mitral valve (MV) E-wave (cm/s)	0.7 (0.6, 0.8)
MV A-wave (cm/s)	0.6 (0.5, 0.7)
MV duration A-wave (ms)	133 (121, 147)
MV deceleration (dec.) Time (ms)	179 (157, 203)
E/e ratio	5.9 (4.9, 7.1)
RV structural echocardiographic parameters	
Right ventricle (RV) (cm)	2.4 (2.1, 2.8)
Right ventricular outflow tract (RVOT) (cm)	3.4 (3.0, 3.7)
RV systolic functional echocardiographic parameters	
Tricuspid annular plane systolic excursion (TAPSE) (cm)	2.4 (2.1, 2.6)
Pulmonary valve (PV) acc. time (ms)	134 (115, 152)
PV acc. slope	5.8 (4.9, 7.1)
hArg (µmol/L)	

**Table 2:** The relation between hArg and cardiac structure and function. The outcomes were analyzed by linear regression adjusted for sex, age, BMI, and eGFR. Bold lettering indicated *p* < 0.05.

	β for 1 SD Decrease of hArg (95% CI)	*p*
LV structural echocardiographic parameters		
LVM (g)	1.29	0.0677
	(0.09, 2.67)	
LVMI (g/m^2^)	0.63	0.0703
	(0.05, 1.31)	
**LVD (mm)**	**0.34**	**<0.0001**
	**(0.19, 0.50)**	
**LVS (mm)**	**0.38**	**<0.0001**
	**(−0.22, 0.54)**	
PWD (mm)	−0.01	0.5514
	(−0.06, 0.03)	
**RWT**	**−0.003**	**0.011**
	**(−0.006, −0.001)**	
Left atrium (mm)	−0.07	0.3795
	(0.09, 0.24)	
Aorta (mm)	0.07	0.2396
	(−0.05, −0.19)	
LV systolic functional echocardiographic parameters		
**LVEF (%)**	**−0.47**	**0.0043**
	**(−0.79, −0.15)**	
**Fractional shortening (%)**	**−0.35**	**0.0141**
	**(−0.62, −0.07)**	
LV diastolic functional echocardiographic parameters		
MV E-wave (mm/s)	−0.002	0.2607
	(−0.008, 0.002)	
MV A-wave (mm/s)	−0.003	0.1708
	(−0.008, 0.001)	
MV duration A-wave (ms)	0.41	0.2844
	(−0.34, 1.17)	
MV dec. time (ms)	0.24	0.7166
	(−1.08, 1.57)	
E/e ratio	−0.02	0.4205
	(−0.08, 0.04)	
RV structural echocardiographic parameters		
RV (mm)	−0.0006	0.9422
	(−0.02, 0.02)	
RVOT (cm)	0.01	0.07
	(−0.001, 0.03)	
RV systolic functional echocardiographic parameters		
TAPSE (cm)	−0.009	0.1772
	(−0.023, 0.004)	
PV acc. time (ms)	0.39	0.3755
	(−0.48, 1.26)	
PV acc. slope	−0.04	0.1977
	(−0.12, 0.02)	

## References

[1] Morris S.M. (2016). Arginine Metabolism Revisited. J. Nutr..

[2] Davids M., Ndika J.D., Salomons G.S., Blom H.J., Teerlink T. (2012). Promiscuous activity of arginine: Glycine amidinotransferase is responsible for the synthesis of the novel cardiovascular risk factor homoarginine. FEBS Lett..

[3] Moali C., Boucher J.L., Sari M.A., Stuehr D.J., Mansuy D. (1998). Substrate specificity of NO synthases: Detailed comparison of l-arginine, homo-l-arginine, their *N* omega-hydroxy derivatives, and *N* omega-hydroxynor-l-arginine. Biochemistry.

[4] Tommasi S., Elliot D.J., Da Boit M., Gray S.R., Lewis B.C., Mangoni A.A. (2018). Homoarginine and inhibition of human arginase activity: Kinetic characterization and biological relevance. Sci. Rep..

[5] Marz W., Meinitzer A., Drechsler C., Pilz S., Krane V., Kleber M.E., Fischer J., Winkelmann B.R., Böhm B.O., Ritz E. (2010). Homoarginine, cardiovascular risk, and mortality. Circulation.

[6] Pilz S., Edelmann F., Meinitzer A., Gelbrich G., Doner U., Dungen H.D., Tomaschitz A., Kienreich K., Gaksch M., Duvinage A. (2014). Associations of methylarginines and homoarginine with diastolic dysfunction and cardiovascular risk factors in patients with preserved left ventricular ejection fraction. J. Card. Fail..

[7] Faller K.M.E., Atzler D., McAndrew D.J., Zervou S., Whittington H.J., Simon J.N., Tomaschitz A., Kienreich K., Gaksch M., Duvinage A. (2018). Impaired cardiac contractile function in arginine: Glycine amidinotransferase knockout mice devoid of creatine is rescued by homoarginine but not creatine. Cardiovasc. Res..

[8] Choe C.U., Atzler D., Wild P.S., Carter A.M., Boger R.H., Ojeda F., Simova O., Stockebrand M., Lackner K., Nabuurs C. (2013). Homoarginine levels are regulated by l-arginine: Glycine amidinotransferase and affect stroke outcome: Results from human and murine studies. Circulation.

[9] Pilz S., Meinitzer A., Tomaschitz A., Drechsler C., Ritz E., Krane V., Wanner C., Boehm B.O., März W. (2011). Low homoarginine concentration is a novel risk factor for heart disease. Heart.

[10] Pilz S., Teerlink T., Scheffer P.G., Meinitzer A., Rutters F., Tomaschitz A., Drechsler C., Kienreich K., Nijpels G., Stehouwer C.D. (2014). Homoarginine and mortality in an older population: The Hoorn study. Eur. J. Clin. Investig..

[11] Drechsler C., Meinitzer A., Pilz S., Krane V., Tomaschitz A., Ritz E., März W., Wanner C. (2011). Homoarginine, heart failure, and sudden cardiac death in haemodialysis patients. Eur. J. Heart Fail..

[12] Atzler D., Rosenberg M., Anderssohn M., Choe C.U., Lutz M., Zugck C., Böger R.H., Frey N., Schwedhelm E. (2013). Homoarginine—An independent marker of mortality in heart failure. Int. J. Cardiol..

[13] Volzke H., Alte D., Schmidt C.O., Radke D., Lorbeer R., Friedrich N., Aumann N., Lau K., Piontek M., Born G. (2011). Cohort profile: The study of health in Pomerania. Int. J. Epidemiol..

[14] World Health Organization (1995). Physical Status: The Use and Interpretation of Anthropometry.

[15] Levey A.S., Coresh J., Greene T., Marsh J., Stevens L.A., Kusek J.W., Van Lente F. (2007). Chronic Kidney Disease Epidemiology Collaboration. Expressing the Modification of Diet in Renal Disease Study equation for estimating glomerular filtration rate with standardized serum creatinine values. Clin. Chem..

[16] Atzler D., Schwedhelm E., Nauck M., Ittermann T., Böger R.H., Friedrich N. (2014). Serum reference intervals of homoarginine, ADMA, and SDMA in the Study of Health in Pomerania. Clin. Chem. Lab. Med..

[17] Volzke H., Haring R., Lorbeer R., Wallaschofski H., Reffelmann T., Empen K., Rettig R., John U., Felix S.B., Dörr M. (2010). Heart valve sclerosis predicts all-cause and cardiovascular mortality. Atherosclerosis.

[18] Schiller N.B., Shah P.M., Crawford M., DeMaria A., Devereux R., Feigenbaum H., Gutgesell H., Reichek N., Sahn D., Schnittger I. (1989). Recommendations for quantitation of the left ventricle by two-dimensional echocardiography. American Society of Echocardiography Committee on Standards, Subcommittee on Quantitation of Two-Dimensional Echocardiograms. J. Am. Soc. Echocardiogr..

[19] Reichek N., Devereux R.B. (1981). Left ventricular hypertrophy: Relationship of anatomic, echocardiographic and electrocardiographic findings. Circulation.

[20] Devereux R.B., Alonso D.R., Lutas E.M., Gottlieb G.J., Campo E., Sachs I., Reichek N. (1986). Echocardiographic assessment of left ventricular hypertrophy: Comparison to necropsy findings. Am. J. Cardiol..

[21] Verbraecken J., Van de Heyning P., De Backer W., Van Gaal L. (2006). Body surface area in normal-weight, overweight, and obese adults. A. comparison study. Metabolism.

[22] Lang R.M., Badano L.P., Mor-Avi V., Afilalo J., Armstrong A., Ernande L., Flachskampf F.A., Foster E., Goldstein S.A., Kuznetsova T. (2015). Recommendations for cardiac chamber quantification by echocardiography in adults: An update from the American Society of Echocardiography and the European Association of Cardiovascular Imaging. Eur. Heart J. Cardiovasc. Imaging.

[23] Stone C.J., Koo C.-Y. (1985). Additive splines in statistics. Proc. Stat. Comput. Sect. Am. Stat. Assoc..

[24] Atzler D., Schwedhelm E., Choe C.U. (2015). l-homoarginine and cardiovascular disease. Curr. Opin. Clin. Nutr. Metab. Care.

[25] Lygate C.A., Schneider J.E., Neubauer S. (2013). Investigating cardiac energetics in heart failure. Exp. Physiol..

[26] Cullen M.E., Yuen A.H., Felkin L.E., Smolenski R.T., Hall J.L., Grindle S., Miller L.W., Birks E.J., Yacoub M.H., Barton P.J. (2006). Myocardial expression of the arginine: Glycine amidinotransferase gene is elevated in heart failure and normalized after recovery: Potential implications for local creatine synthesis. Circulation.

[27] Seppala I., Oksala N., Jula A., Kangas A.J., Soininen P., Hutri-Kahonen N., März W., Meinitzer A., Juonala M., Kähönen M. (2017). The biomarker and causal roles of homoarginine in the development of cardiometabolic diseases: An observational and Mendelian randomization analysis. Sci. Rep..

[28] Atzler D., McAndrew D.J., Cordts K., Schneider J.E., Zervou S., Schwedhelm E., Neubauer S., Lygate C.A. (2017). Dietary Supplementation with Homoarginine Preserves Cardiac Function in a Murine Model of Post-Myocardial Infarction Heart Failure. Circulation.

[29] Atzler D., Schönhoff M., Cordts K., Ortland I., Hoppe J., Hummel F.C., Gerloff C., Jaehde U., Jagodzinski A., Böger R.H. (2016). Oral supplementation with l-homoarginine in young volunteers. Br. J. Clin. Pharmacol..

